# The tip of the iceberg: finding patients with heart failure with preserved ejection fraction in primary care. An observational study

**DOI:** 10.3399/bjgpopen18X101606

**Published:** 2018-09-19

**Authors:** Christi Deaton, Duncan Edwards, Alexandra Malyon, M Justin S Zaman

**Affiliations:** 1 Florence Nightingale Foundation Clinical Professor of Nursing, Department of Public Health and Primary Care, University of Cambridge School of Clinical Medicine, Cambridge, UK; 2 NIHR Doctoral Research Fellow, Department of Public Health and Primary Care, University of Cambridge School of Clinical Medicine, Cambridge, UK; 3 Clinical Research Development Nurse, Department of Education and Research, Cambridge University Hospital, Cambridge, UK; 4 Consultant Cardiologist, Department of Medicine, James Paget University Hospital, Gorleston-on-Sea, UK

**Keywords:** heart failure with preserved ejection fraction, diagnosing heart failure, echocardiogram, heart failure, diagnostic tests, delayed diagnosis

## Abstract

**Background:**

Heart failure with preserved ejection fraction (HFpEF) is under-identified in primary care.

**Aim:**

The aim of this study was to determine what information is available in patients’ primary care practice records that would identify patients with HFpEF.

**Design & setting:**

Record review in two practices in east of England.

**Method:**

Practices completed a case report form on each patient on the heart failure register and sent anonymised echocardiography reports on patients with an ejection fraction (EF) >50%. Reports were reviewed and data analysed using SPSS (version 25).

**Results:**

One hundred and forty-eight patients on the heart failure registers with mean age 77 +12 years were reviewed. Fifty-three patients (36%) had possible HFpEF based on available information. These patients were older and multimorbid, with a high prevalence of overweight and obesity. Confirmation of diagnosis was not possible as recommended HFpEF diagnostic information (natriuretic peptides, echocardiogram parameters of structural heart disease and diastolic function) was widely inconsistent or absent in these patients.

**Conclusion:**

Without correct identification of HFpEF, patient management may be suboptimal or inappropriate, and lack the needed focus on comorbidities and lifestyle that can improve patient outcomes. This study describes in detail the characteristics of many of the patients who probably have HFpEF in a real-world sample, and the improvements and diagnostic information required to better identify them. Identifying more than the tip of the iceberg that is the HFpEF population will allow the improvement of the quality of their management, the prevention of ineffective health care, and the recruitment of patients into research.

## How this fits in

Patients with HFpEF comprise half of the patients with heart failure but are under-identified in primary care. Patients with possible HFpEF seldom had natriuretic peptides measured, and lacked consistent echocardiographic measurement of relevant parameters for structural heart disease and diastolic function needed for diagnosis. The absence of relevant diagnostic information and interpretation in primary care hinders identification and appropriate management of patients with HFpEF.

## Introduction

Heart failure affects 900 000 people in the UK and accounts for 2% of NHS expenditure, primarily due to emergency hospitalisations.^1,2^ Many clinicians in general practice may be surprised to know that approximately half of these patients have a type of heart failure called heart failure with a preserved ejection fraction (HFpEF), as very few of these patients ever receive a specific diagnosis.^3–7^ This really matters, as HFpEF patients need a different management strategy to patients with heart failure with reduced ejection fraction (HFrEF, where the heart ejects <40% of its volume of blood each time it contracts). Patients with HFpEF need a focus on regulation of fluid status with diuretics, self-management including diet and exercise, and control of comorbid conditions such as atrial fibrillation (AF) and hypertension, rather than drugs and devices which are the mainstay for HFrEF.^8,9^ Misdiagnosis also undermines quality improvement and research into HFpEF. Misdiagnosis would no longer be tolerated in fields such as diabetes and stroke, where specific diagnosis is understood to be vital for patient care, and should no longer be tolerated in the field of heart failure.

However, diagnosing heart failure without a reduced left ventricular ejection fraction (LVEF) is more complex and this complexity likely stems from an incomplete understanding of the exact pathophysiological processes leading to HFpEF, changes in and lack of agreement on diagnostic criteria, a heterogeneous population with HFpEF, a lack of specific evidence-based treatment, and a focus on HFrEF for whom evidence-based treatment exists.^5,8,10–14^ The recent European Society of Cardiology (ESC) guidelines on acute and chronic heart failure^8^ define HFpEF as symptoms and signs of heart failure, an LVEF >50%, elevated levels of natriuretic peptides (brain natriuretic peptide >35 picograms per millilitre [pg/mL] or N-terminal pro b-type natriuretic peptide [NT-ProBNP] >125 pg/mL) and at least one of the following additional criteria: relevant structural heart disease (left ventricular hypertrophy [LVH] and/or left atrial enlargement; or diastolic dysfunction. These criteria pose some challenges for primary care practitioners. Although recommended to rule out heart failure by the ESC and NICE, not all patients presenting with suspected heart failure in primary care have natriuretic peptides measured. LVH and left atrial enlargement are easily and frequently assessed but other recommended echocardiographic parameters for HFpEF are more technically challenging and require specialist interpretation. Diastolic dysfunction is defined by specific indices on echocardiogram measuring mitral valve early and late diastolic inflow, early diastolic tissue velocity, the ratio between early mitral inflow velocity and mitral annular early diastolic velocity and peak tricuspid regurgitation velocity.^8,15^ Although not a diagnostic parameter, elevated pulmonary artery systolic pressure (PASP) is common, and is an important indicator for prognosis and management. Recommendations for what to measure on echocardiogram and values considered abnormal differ between professional groups (Box 1).

**Box 1.** Recommended echocardiographic indices for diagnosing HFpEF and diastolic dysfunction


**European Society of Cardiology clinical practice guidelines on acute and chronic heart failure 2016^3^**
Relevant structural heart disease Left ventricular hypertrophy (left ventricular mass index >115 g/m^2^ for males and >95 g/m^2^ for females) Left atrial enlargement (left atrial volume index >34 mL/m^2^) Diastolic dysfunction Early diastolic tissue velocity (e’ mean septal-lateral <9 cm/sec) Ratio between early mitral inflow velocity and mitral annular early diastolic velocity (E/e’>13) Deceleration time (DecT) of mitral valve early diastolic inflow (MV-E) m/sec E/A ratio <1 or >2 Isovolumetric relaxation time (IVRT)
**American Society of Echocardiography and the European Association of Cardiovascular Imaging 2016^9^**
Annular e’ velocity Septal e’ <7 cm/sec Lateral e’ <10 cm/sec Average E/e’ ratio >14 Left atrium maximum volume index (>34 mL/m^2^) Peak tricuspid regurgitation velocity >2.8 m/sec Note: LV diastolic function is normal if more than half of the variables do not meet the cut-off for identifying abnormal function.

cm/sec = centimetres per second. E = early mitral diastolic inflow. e’ = early diastolic tissue velocity. E/A = ratio between peak early (E) and late (A) diastolic filling velocities. E/e’ = ratio between early mitral inflow velocity and mitral annular early diastolic velocity. g/m^2^ = grams per metre squared. LV = left ventricle. m/sec = metres per second. mL/m^2^ = millilitres per metre squared. MV = mitral valve.

Primary care provides sole or shared care for all patients with heart failure in the UK, and maintains registers of heart failure patients as mandated by the Quality Outcomes Framework (QOF). QOF also requires that an echocardiogram is done to confirm or refute the heart failure diagnosis. The aim of this study was to determine what information was available in patients’ full primary care practice records to identify patients with HFpEF from within practice heart failure patient registers.

## Method

Two primary care practices in the east of England were recruited for the study; one urban and one in a smaller town accessing echocardiography services run by different hospitals. Each practice completed a simple case report form (CRF) on each patient on the heart failure register of the practice requesting brief demographic and clinical information. Data were collected during 2016–17, but included patients on heart failure registers regardless of when diagnosed. Practices were asked to send anonymised echocardiography reports on all patients with an EF >50%, or those patients with LVEF labelled as ‘normal, preserved, or near-normal’. The echocardiography reports or letters detailing echocardiographic information were scrutinised for parameters measured and assessment of left ventricular diastolic function.

Data were entered into an SPSS (version 25) database by the research nurse and re-checked by the principal investigator. The principal investigator and a consultant cardiologist reviewed the data and echocardiographic reports and letters on each patient. Patients were divided into three groups based on EF as defined by the ESC:^8^ EF <40% (HFrEF or LVSD), EF 40–49% as heart failure with a mid-range EF (HFmrEF), and EF >50% (HFpEF). Patients were included in the HFrEF category if their systolic function was described as severely impaired even if a numeric value was not provided. Similarly, patients were included in the HFpEF category if their EF was described as normal, near-normal, or preserved. A fourth group comprised those patients for whom echocardiographic data were missing or not clear enough for categorisation. This final group included those with descriptions of mild, moderate, or mild–moderate systolic dysfunction as there was uncertainty regarding matching these descriptors to a specific numerical LVEF. These groups were compared on demographic and clinical characteristics.

### Results

The two practices participating in this study differed in size and socioeconomic deprivation of the practice area. The urban practice had a list size of 7890, with 48 patients on the heart failure register, and was in the least deprived decile for socioeconomic status. The town practice had a list size of 13 229 with 100 patients on the heart failure register, and was in the fifth most deprived decile. The proportion of patients aged ≥65 were similar (15–17%) and both practices had predominantly white patients. Non-white ethnicity ranged from 5% (in the smaller town practice) to 19% (urban).

CRFs were completed on 148 patients aged 40–99 years. Patients had a mean age of 77 +12 years with multiple comorbid conditions and a high prevalence of overweight and obesity (Table 1). Echocardiogram reports or letters were unavailable or did not provide enough information to characterise 31 patients (21%) by EF. This 21% included patients placed on the heart failure register prior to the QOF requirement for echocardiographic confirmation of heart failure, patients awaiting echocardiography, echocardiograms reported as technically difficult with limited data, and a few with unknown reasons for lack of echocardiogram reports. Sixty-nine of the patients (56% of those with an echocardiogram) had a numeric EF provided, and the other reports used verbal descriptions (for example, preserved or moderately impaired) to describe systolic function and EF.

**Table 1.** Characteristics of sample

	Patients on heart failure registers (*n* = 148)
Age, years	76.9 + 12
Duration of heart failure diagnosis, years	5.3 + 4
Female sex, %	39
Hypertension, %	84
Chronic kidney disease, %	44
Atrial fibrillation, %	41
BMI 25–29.9 kg/m^2^,%	31
BMI >30 kg/m^2^, %	39
Ischaemic heart disease, %	32
Diabetes, %	25
Valvular heart disease, %	22
COPD, %	16
Stroke, %	15
Asthma, %	10
Current smoker, %	10
Ex-smoker, %	30
Echocardiogram information available, %	79

COPD = chronic obstructive pulmonary disease, HF = heart failure; kg/m^2^ = kilograms per metre squared

Grouping patients by EF resulted in 43 patients (29%) with EF <40% or labelled severe systolic dysfunction or severely impaired EF; 21 (14%) with EF 40–49%; 53 (36%) with EF >50% or described as having normal, near-normal, or preserved EF; and 31 (21%) missing or unable to be categorised. Small sample sizes in the groups precluded finding significant differences in characteristics by EF group except for a lower recorded prevalence of atrial fibrillation in patients for whom echocardiographic data were missing or unclear (Table 2). Patients with EF >50% had a mean age of nearly 80, 83% had hypertension, 81% were overweight or obese, 65% had more than 3 comorbid conditions, half had chronic kidney disease, 45% were women, and nearly one-third had diabetes. Although not significant, there were trends toward older age, a higher proportion of women, greater prevalence of obesity and multiple comorbidities, and lower rates of ischaemic heart disease in patients with EF >50% compared to those with EF <40%. Only 9% of the total sample had natriuretic peptides (specifically NT-ProBNP) results available in the record. Six of the 53 patients with an EF >50% had natriutric peptides measured, with 5 of these being elevated well above the ESC guideline recommended level for considering a diagnosis of heart failure (NT-ProBNP >125 pg/mL).^7^ Seven of the 43 patients with EF <40% had NT-ProBNP results. The mean NT-ProBNP for those with EF >50% was 2699 +2138 pg/mL, compared to 4858 +6479 pg/mL for those with EF <40%.

**Table 2.** Clinical characteristics by EF group

Characteristics	EF <40% (*n* = 43)	EF 40–49% (*n* = 21)	EF >50% (*n* = 53)	Unclear or missing (*n* = 31)	*P* value
Mean age (SD)	76 (13)	76 (13)	79.8 (11)	74.2 (11)	0.161
Female sex, %	30	29	45	46	0.293
Hypertension, %	80	81	83	93	0.442
IHD, %	39	29	30	29	0.743
CKD, %	39	38	51	42	0.615
Diabetes, %	24	10	32	26	0.254
AF, %	49	43	47	19	0.048
COPD, %	12	10	19	19	0.638
Stroke, %	15	14	17	13	0.964
Valvular heart disease, %	24	24	28	7	0.123
BMI 25–29.9 kg/m^2^, %	33	18	34	30	0.630
BMI >30 kg/m^2^, %	33	29	47	40	0.503
>3 comorbidities, %	51	41	65	63	0.270
Mean duration of HF, years (SD)	4.3 (4.5)	5.9 (4.4)	5.0 (4.0)	6.9 (5.0)	0.08

AF = atrial fibrillation. BMI = body mass index. CKD = chronic kidney disease. COPD = chronic obstructive pulmonary disease. EF = ejection fraction. HF = heart failure. IHD = ischaemic heart disease. Kg/m^2^ = kilograms per metre squared. SD = standard deviation.

### Echocardiographic data

Of the 53 patients with documented EF >50%, 39 were found to have echocardiographic reports that included at least one of the parameters recommended for diagnosing HFpEF and diastolic dysfunction (Table 3). Left ventricular size and mass were commented on in 35 and left atrial size and volume were discussed in 33. Sixteen of 35 patients (46%) had at least some degree of LVH, and the left atrium was dilated in 28 of 33 patients (85%). Only in seven of the 39 patients (18%) were both the left atrium and left ventricle normal. At least one measure of diastolic function was available in 24 patients and 17 had at least one abnormal measure. Comments about diastolic function were found in reports of 12 patients, with 11 of these patients having some degree of diastolic dysfunction noted, and one with elevated filling pressures. However measurements of specific indices of diastolic function were not documented in five patients with diastolic dysfunction noted on the report.

**Table 3.** Echocardiographic data in 39 patients with EF >50%

	EF >50%^a^
Mean EF (SD)	57 +5%
At least one recommended measure of structural heart disease documented, *n/N* (%)	35/39 (90)
LVH present, *n/N* (%)	16/35 (46)
Concentric LVH, *n/N* (%)	6/16 (38)
LA dilated, *n/*N (%)	28/33 (85)
Both LV and LA normal *n/N* (%)	7/39 (18)
At least one recommended index of diastolic function measured, *n/N* (%)	24/39 (62)
E/A ratio, *n/N* (%)	13/24 (54)
e' lateral, *n/N* (%)	5/24 (21)
e' septal, *n/N* (%)	5/24 (21)
E/e' mean, n/N (%)	19/24 (79)
TRV, *n/N* (%)	14/24 (58)
At least one recommended index of diastolic function is abnormal, *N/n* (%)	17/24 (71)
Number of diastolic function indices that are abnormal	
One	8
Two	5
Three	3
Four	1
Diastolic dysfunction labelled on report or in letter where diastolic function mentioned, *n/N* (%)	11/20 (55)
RV dysfunction present, *n/N* (%)	7/31 (23)
RV dilation, *n/N* (%)	9/27 (33)
PH documented, *n/N* (%)	7/26 (27)

^a^includes patients labelled as having a ‘normal’, ‘near-normal’ or ‘preserved’ EF

E/A = ratio between peak early (E) and late (A) diastolic filling velocities. EF = ejection fraction. e’ = early diastolic tissue velocity. E/e’ = ratio between early mitral inflow velocity and mitral annular early diastolic velocity. LA = left atrium. LV = left ventricle. LVH = left ventricular hypertrophy. PH = pulmonary hypertension. RV = right ventricle. TRV = peak tricuspid regurgitation velocity.

In total 24 patients had both measures of relevant structural heart disease and diastolic function, and 15 patients had abnormal values of both of these. Seven patients had pulmonary hypertension (PH) or possible PH documented, and PASPs were available in 9 patients. Differences on echocardiogram reports in measurements and information provided for patients with possible HFpEF differed by individual echocardiographers, more so than by the service. This finding was independent of reports of technical or other difficulties in performing the echocardiogram.

### Diagnosis

Very few patients would have met the ESC diagnostic criteria for HFpEF given the lack of recommended measurements. Although 73.5% of the 53 patients with possible HFpEF had at least one measure of structural heart disease and/or diastolic function assessed, there was a lack of consistency in which indices were measured and how many were reported. Natriuretic peptide levels were available in <10% of all of the patients.

## Discussion

### Summary

In two primary care registers of heart failure patients, this study found that patients with possible HFpEF comprised 36% of the patients. This group of patients were on average a few years older than the other heart failure patient groups, and had a high comorbidity burden including the highest prevalence of being overweight or obese. Confirmation of diagnosis was not possible as precise HFpEF diagnostic information was widely inconsistent or absent in these patients. Echocardiographic indices related to diastolic function (and to a less extent structural heart disease), and interpretation of findings related to HFpEF were extremely limited or missing. An additional 21% of patients had missing echocardiograms or were unable to be categorised by EF, and this may include additional patients with HFpEF. Natriuretic peptide results were also infrequently available.

### Strengths and limitations

The strength of the study was in the thorough review of anonymised echocardiographic reports and cCRFs from patients on the heart failure registers of two primary care practices in different areas, and comparison with recommended criteria for diagnosing HFpEF. However the study did not include a search for patients with heart failure who may not have been on the heart failure registers. Heart failure registers have been found to have varying levels of accuracy (Audit British Heart Foundation, Bury Heart Failure Audit, unpublished data, 2010) and patients may be placed on the heart failure register prior to confirmation. A further limitation of the study is that there were a limited number of patients and both practices were in the east of England with limited ethnic diversity.

### Comparison with existing literature

Patients with HFpEF comprise half of the patients with heart failure, and epidemiological analyses have shown an increasing prevalence of patients with HFpEF especially among those referred to acute services from the community.^16^ Given the mean age of the patients on the two heart failure registers (77 years) in this study, a higher proportion of patients with possible HFpEF would be expected. A pooled analysis of 105 studies with 196 105 patients with undifferentiated heart failure recruited from general practice indicated that the predominant phenotype was an older woman with hypertension rather than ischaemic heart disease,^17^ which would be suggestive of a high prevalence of HFpEF. The patients with possible HFpEF in the current analysis had a non-significant trend toward older age, a higher proportion of women, and multiple comorbidities. The increasing prevalence of HFpEF among older patients suggests that only the tip of a potential iceberg of patients with HFpEF in primary care is being identified.

Both underdiagnosis and overdiagnosis of heart failure in primary care have been found in other studies.^6,18,19^ Various routes to diagnosis of heart failure in patients in UK primary care have been documented, with nearly 80% of patients being diagnosed in secondary care, and less than one quarter following the recommended NICE diagnostic pathway.^20^ A recent survey found that most GPs (84%) did not diagnose HFpEF, and only 7% were very confident in their ability to do so.^21^ Although echocardiography is an essential tool for determining the diagnosis and type of heart failure it has been found to be underused in general practice. In a study of 683 patients with a heart failure diagnosis in 30 general practices, only 45.2% had undergone an echocardiogram at the start of the study in 2010–2011.^18^ Munoz and colleagues^22^ analysed records from 8376 patients with diagnoses of heart failure in 52 primary care practices in Barcelona 2009–2012. The majority of patients (91.5%) did not have an available EF. Most of the patients in the present analysis had an echocardiogram done, which may be due to the influence of the QOF criteria. However many GPs in the UK lack confidence in interpretation of echocardiography reports from open access services, especially in regards to HFpEF.^21^

Although echocardiogram reports or letters describing results were available for the majority of patients in our study, data on echocardiographic indices to diagnose HFpEF were inconsistently and infrequently measured in patients with possible HFpEF. Guidelines such as the ESC specify what needs to be measured for HFpEF assessment, but disagreement regarding criteria exists. The ESC guidelines^8^ do not specify how many measures of diastolic function need to be abnormal to establish a diagnosis of diastolic dysfunction, but the American Society of Echocardiography and the European Association of Cardiovascular Imaging^15^ require at least half of 5 recommended parameters to be abnormal. The British Society of Echocardiography (BSE) minimum dataset includes a few parameters related to diastolic function, and they have also published a practical guide on assessment and grading of diastolic dysfunction. The BSE noted that confidence in assessing and grading diastolic dysfunction increases with increasing numbers of corroborative parameters but does not specify how many.^23,24^ The inconsistency in reports seen in this analysis could also reflect local and regional practice and guidance, limited time for echocardiography appointments in busy services, and limited patient information provided in referral. The inconsistency in reporting specific parameters varied by echocardiographers within the two services rather than by service.

Other important characteristics and prognostic indicators such as pulmonary artery pressures were also infrequently documented in this sample of patients. In a community sample of 244 patients with HFpEF, elevated PASPs were found in 83% of patients,^25^ and higher PASP was associated with mortality (age-adjusted hazard ratio 1.22 per 10 mm Hg; *P*<0.005). Another analysis (1663 patients with heart failure) found an elevated PASP >40 mm Hg to be an independent predictor of survival in patients with heart failure and an EF >40% with a hazard ratio of 2.27 (95% confidence intervals = 1.58 to 3.26; *P*<0.001).^26^

### Implications for practice

The lack of consistent and relevant information and interpretation of findings from echocardiography and other diagnostic tests for patients with suspected heart failure could lead to misdiagnosis and inappropriate treatment. Patients with HFpEF may have reason to be on similar medications to those with HFrEF for control of hypertension and other cardiac conditions, but these should not be prescribed automatically as HFpEF-specific treatment due to lack of efficacy in improving mortality and morbidity. Some healthcare professionals may argue that, until there are evidence-based treatments for management of HFpEF, a formal diagnosis of HFpEF is unnecessary. However there is a clear message that HFrEF and HFpEF are not the same, that clinicians must treat HFpEF now by managing comorbidities, and that the greatest reductions in mortality and morbidity may result from treating comorbidities.^9,14^ In the CHARM clinical trial with over 1000 patients with HFpEF, the burden of non-cardiac conditions accounted for a greater proportion of risk for death than cardiac burden (population attributable risk 49% versus 15%; *P*<0.05).^27^ Comorbid conditions have a greater impact on functional class and physical health status in HFpEF, and hospitalisations and readmissions for non-cardiac causes are higher in patients with HFpEF compared to HFrEF.^28,29^ An analysis of over 43 000 patients hospitalised for HFpEF in the US found a 1-year composite of mortality and all-cause readmission to be 74%.^30^

Patients with HFpEF will also benefit from a focus on self-management and lifestyle factors. Physical activity has been shown to improve fitness and quality of life in patients with HFpE,^31^ and emerging evidence indicates that weight loss may also improve outcomes in obese patients.^32^ Patients experiencing problems with fluid overload may benefit from restrictions in fluid and salt intake. Diet non-compliance was shown to be a precipitating factor in hospitalisation for heart failure regardless of LVEF, and in another analysis patients with HFpEF who received sodium-restriction dietary instruction at time of hospital discharge had significantly lower risk of 30-day combined readmission and death.^33,34^

In conclusion, the findings of this study have highlighted that there is a deficit in identification of patients with HFpEF. The implications for this are that without correct identification, patient management may be suboptimal or inappropriate, and lack the needed focus on comorbidities and lifestyle that can improve patient outcomes. Furthermore, without correct identification patients cannot be recruited into clinical trials and other studies that could develop and test HFpEF-specific therapies. The authors have described in detail the characteristics of many of the patients who probably have HFpEF in a real-world sample, and the improvements and diagnostic information (comprehensive echocardiogram reports and natriuretic peptides) required to better identify them. Identifying more than the tip of the iceberg that is the HFpEF population will allow the improvement of the quality of their management, the prevention of ineffective health care, and the recruitment of patients into research.

## References

[1] NICE, Royal College of Physicians (UK) (2010). Chronic heart failure: National Clinical Guideline for diagnosis and management in primary and secondary care [full version of the NICE clinical guideline No 108].

[2] Donkor A, Cleland J, McDonagh T, National Heart Failure Audit Steering Group (2014). National Heart Failure Audit.

[3] Lam CS, Donal E, Kraigher-Krainer E (2011). Epidemiology and clinical course of heart failure with preserved ejection fraction. Eur J Heart Fail.

[4] Borlaug BA (2014). The pathophysiology of heart failure with preserved ejection fraction. Nat Rev Cardiol.

[5] Banerjee P (2016). Heart failure with preserved ejection fraction: A clinical crisis. Int J Cardiol.

[6] Boonman-de Winter LJ, Rutten FH, Cramer MJ (2012). High prevalence of previously unknown heart failure and left ventricular dysfunction in patients with type 2 diabetes. Diabetologia.

[7] Clark AM, Flynn R, Hsu ZY (2013). Heart failure with preserved ejection fraction: health services implications of a stealth syndrome. Eur J Cardiovasc Nurs.

[8] Ponikowski P, Voors AA, Anker SD, ESC Scientific Document Group (2016). 2016 ESC Guidelines for the diagnosis and treatment of acute and chronic heart failure: The Task Force for the diagnosis and treatment of acute and chronic heart failure of the European Society of Cardiology (ESC). Developed with the special contribution of the Heart Failure Association (HFA) of the ESC. Eur Heart J.

[9] Shah SJ, Gheorghiade M (2008). Heart failure with preserved ejection fraction: treat now by treating comorbidities. JAMA.

[10] Redfield MM (2017). Heart failure with preserved ejection fraction. N Engl J Med.

[11] Fuat A, Hungin AP, Murphy JJ (2003). Barriers to accurate diagnosis and effective management of heart failure in primary care: qualitative study. BMJ.

[12] Paulus WJ (2016). Turning the retrospectroscope on heart failure with preserved ejection fraction. J Card Fail.

[13] Hatle L (2007). How to diagnose diastolic heart failure a consensus statement. Eur Heart J.

[14] Oktay AA, Shah SJ (2015). Diagnosis and management of heart failure with preserved ejection fraction: 10 key lessons. Curr Cardiol Rev.

[15] Nagueh SF, Smiseth OA, Appleton CP (2016). Recommendations for the evaluation of left ventricular diastolic function by echocardiography: an update from the American Society of Echocardiography and the European Association of Cardiovascular Imaging. J Am Soc Echocardiogr.

[16] Owan TE, Hodge DO, Herges RM (2006). Trends in prevalence and outcome of heart failure with preserved ejection fraction. N Engl J Med.

[17] Smeets M, Henrard S, Aertgeerts B (2018). Methods to identify heart failure patients in general practice and their impact on patient characteristics: A systematic review. Int J Cardiol.

[18] Valk MJ, Mosterd A, Broekhuizen BD (2016). Overdiagnosis of heart failure in primary care: a cross-sectional study. Br J Gen Pract.

[19] van Riet EE, Hoes AW, Limburg A (2014). Prevalence of unrecognized heart failure in older persons with shortness of breath on exertion. Eur J Heart Fail.

[20] Bottle A, Kim D, Aylin P (2018). Routes to diagnosis of heart failure: observational study using linked data in England. Heart.

[21] Hancock HC, Close H, Fuat A (2014). Barriers to accurate diagnosis and effective management of heart failure have not changed in the past 10 years: a qualitative study and national survey. BMJ Open.

[22] Muñoz MA, Mundet-Tuduri X, Real J (2017). Heart failure labelled patients with missing ejection fraction in primary care: prognosis and determinants. BMC Fam Pract.

[23] Mathew T, Steeds RP, Jones R (2013). A guideline protocol for the echocardiographic assessment of diastolic dysfunction.

[24] Wharton G, Steeds R, Allen J (2015). A minimum dataset for a standard adult transthoracic echocardiogram: a guideline protocol from the British Society of Echocardiography. Echo Res Pract.

[25] Lam CS, Roger VL, Rodeheffer RJ (2009). Pulmonary hypertension in heart failure with preserved ejection fraction: a community-based study. J Am Coll Cardiol.

[26] Ghio S, Guazzi M, Scardovi AB (2017). Different correlates but similar prognostic implications for right ventricular dysfunction in heart failure patients with reduced or preserved ejection fraction. Eur J Heart Fail.

[27] Wolsk E, Claggett B, Køber L (2018). Contribution of cardiac and extra-cardiac disease burden to risk of cardiovascular outcomes varies by ejection fraction in heart failure. Eur J Heart Fail.

[28] Edelmann F, Stahrenberg R, Gelbrich G (2011). Contribution of comorbidities to functional impairment is higher in heart failure with preserved than with reduced ejection fraction. Clin Res Cardiol.

[29] Chan MM, Lam CS (2013). How do patients with heart failure with preserved ejection fraction die?. Eur J Heart Fail.

[30] Ziaeian B, Heidenreich PA, Xu H (2017). Race/Ethnic Differences in Outcomes Among Hospitalized Medicare Patients With Heart Failure and Preserved Ejection Fraction. JACC Heart Fail.

[31] Pandey A, Parashar A, Kumbhani D (2015). Exercise training in patients with heart failure and preserved ejection fraction: meta-analysis of randomized control trials. Circ Heart Fail.

[32] Kitzman DW, Brubaker P, Morgan T (2016). Effect of caloric restriction or aerobic exercise training on peak oxygen consumption and quality of life in obese older patients with heart failure with preserved ejection fraction: a randomized clinical trial. JAMA.

[33] Kapoor JR, Kapoor R, Ju C (2016). Precipitating clinical factors, heart failure characterization, and outcomes in patients hospitalized with heart failure with reduced, borderline, and preserved ejection fraction. JACC Heart Fail.

[34] Hummel SL, DeFranco AC, Skorcz S (2009). Recommendation of low-salt diet and short-term outcomes in heart failure with preserved systolic function. Am J Med.

